# Quantifying collective identity online from self-defining hashtags

**DOI:** 10.1038/s41598-022-19181-w

**Published:** 2022-09-03

**Authors:** Alexander T. J. Barron, Johan Bollen

**Affiliations:** 1 Luddy School of Informatics, Computing, & Engineering, Indiana University-Bloomington, Bloomington, USA; 2 Cognitive Science Program, Indiana University-Bloomington, Bloomington, USA

**Keywords:** Psychology, Human behaviour, Computer science, Information theory and computation

## Abstract

Mass communication over social media can drive rapid changes in our sense of collective identity. Hashtags in particular have acted as powerful social coordinators, playing a key role in organizing social movements like the Gezi park protests, Occupy Wall Street, *#metoo*, and *#blacklivesmatter*. Here we quantify collective identity from the use of hashtags as self-labels in over 85,000 actively-maintained Twitter user profiles spanning 2017–2019. Collective identities emerge from a graph model of individuals’ overlapping self-labels, producing a hierarchy of graph clusters. Each cluster is bound together and characterized semantically by specific hashtags key to its formation. We define and apply two information-theoretic measures to quantify the strength of identities in the hierarchy. First we measure collective identity coherence to determine how integrated any identity is from local to global scales. Second, we consider the conspicuousness of any identity given its vocabulary versus the global identity map. Our work reveals a rich landscape of online identity emerging from the hierarchical alignment of uncoordinated self-labeling actions.

## Introduction

The nature and structure of collective identity is a driver of a range of present-day socioeconomic phenomena. Societal norms of identity evolve rapidly and continuously, with implications for public policy, politics, and law^1–5^. Questions of identity permeate popular culture presently, and have increasingly been the subject of scholarly and scientific investigations^1,5–7^.

Here we quantitatively study the social construction of collective identity by treating identity as a personal labeling process, a concept explored separately in sociology and psychology through Social Identity Theory and Self-Categorization Theory^8–10^. A central tenet of this approach is the discrete affiliations individuals assign to themselves are references to known, external social categorizations^8,9^. These self-labels allow an individual to express an identity relative to the collections of attributes that others use to express their own identities. From the similarity between individual labeling choices thus emerges a super-structure of user collectives with aligned identity. Under such a conception of collective identity, how does one measure an identity’s “strength” in unity or coherence compared against others? Similarly, but distinctly, how measurably identifiable—or conspicuous—are the qualities of any particular identity against a landscape of identity collectives? With our unprecedented data set and information theoretic approach described in this work, we shed light on these questions.

Social media lends itself well to the process of self-labeling. Many platforms such as Twitter allow individuals to create user profiles to describe themselves, providing a public space to express identities with words or other language tokens. Hashtags are likely candidates for such an identity labeling process. The inclusion of the hash character (#) in any token signals a desire to connect to others who are presumed to understand what the hashtag means in a broader social context. The hashtag is thus an interesting signal for individual identity labeling; it serves as a focal point of common knowledge whose use by any one person implies its use by many others^11^. For this reason, hashtags have arisen as crucial social movement coordinators^1^ that may bear signals of collective identity when used as self-descriptors in users’ online profiles. For example, a user that includes *#metoo* in their profile expresses a facet of their personal identity, expecting others to understand it as an identity token that expresses shared knowledge, implicitly inviting others to do the same. Thus a social construction of individuals with aligned identities is co-created.

## Identity: describing ourselves

We focus on social media profiles as a large-scale, detailed source of data about individuals publicly declaring their identities. The Twitter streaming API has been a valuable resource for research on tweets themselves^12–14^, but hides another resource: user profiles, containing self-descriptions, held in the metadata of each tweet. Due to a quirk in the standard Twitter API (as of 2020), user profile information is provided with every tweet, but their contents reflect the profile at the time of the API request, *not* the time of the tweet. Twitter’s *streaming sample*, in contrast, preserves user profile descriptions *when it delivers* its daily tweet sample, providing a historical record of self-descriptions. Profiles in tweets delivered via streaming sample for a particular user thus comprise a timeline of user self-descriptions. We record each user’s self-representation as a set of hashtags from their profile updates, collected over a nearly 2-year period.

Our user cohort is designed to study those who are active and engaged in maintaining their identities on Twitter. To satisfy these criteria, we start with an initial user pool of nearly 2 million English-language, non-bot users known to have actively maintained their profiles between 2012 and 2014. We harvest the decahose tweets of these same users occurring from August 2017 through December 2019, keeping only the roughly 1.1 million users actively maintaining their profiles in this time frame, establishing multi-year engagement. We filter out non-personal organization accounts and retain only users who share at least one hashtag with another user. Our final collection contains 85,839 non-bot, non-organization users actively engaged in maintaining and updating their self-descriptions (see “Methods” section for details). We specifically end collection before 2020 to avoid any influence from the COVID pandemic.

## Mapping collective identity through self-labeling alignment

To capture groups of individuals with similar self-labeling behavior, yet distinct from other such groups, we create a graph model of shared identity with vertices representing users, and edges between users representing whether they hold hashtags in common. When individuals hold more hashtags in common, their declared identities are more similar; therefore, partitioning the resulting user graph into clusters of dense connections produces groups of users with *aligned* identities. To capture identity alignment from the edge weights of the resulting graph, we partition the graph via modularity-maximization, because of its focus on clusters with high edge concentration^15^. Given a partition of the user graph into clusters, we can leverage our knowledge of the hashtags from which each edge was induced, and the definition of modularity, to rank self-labels by their individual contribution to a given cluster’s modularity score. The top-n ranked self-labels that contribute the most to a cluster’s alignment are deemed “prototypical”, i.e. suitable descriptors of the cluster’s core collective identity (see “Methods” section).

**Figure 1:**
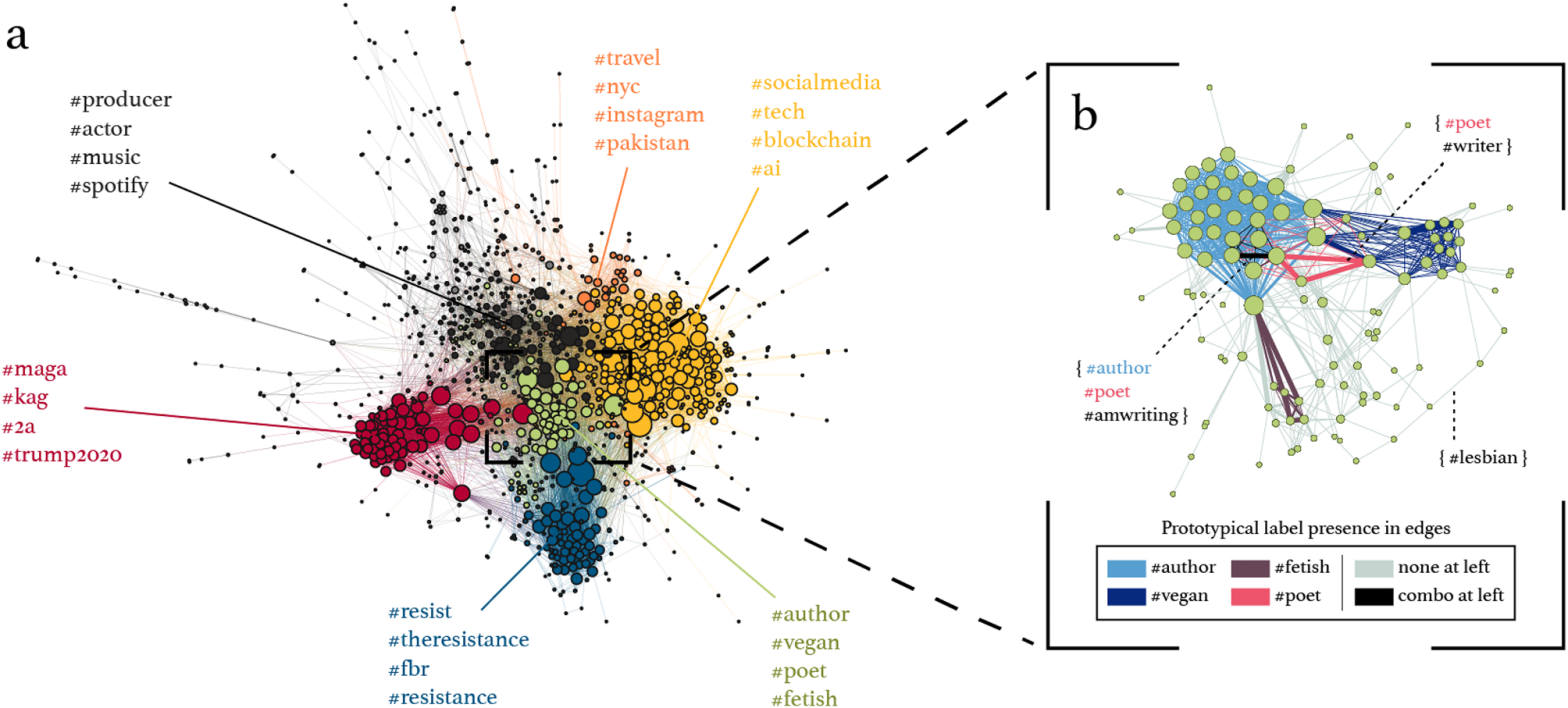
Left: Visualization of user identity-sharing graph for the giant component of the top 2500 most-followed users. Vertices represent users, with size indicating vertex degree; edges represent the presence of commonly-held identity hashtags for the user pair, weighted by the number of tags in common. Modularity-based clusters are represented by color. Semantic annotations indicate the top-4 prototypical hashtags that contributed most to the modularity, and, therefore, to the degree of identity alignment, of each cluster. Right: Detail of the *#author*/*#vegan* identity cluster at left, showing inner structure and composition of self-label co-use in edges. Edge colors here represent the presence of exactly one, a combination, or none of the four prototypical labels for the cluster, illustrating the modularity-derived importance of each label for this complete identity cluster. Dotted lines point to specific edge composition annotations.

To illustrate how the alignment of self-labeling hashtags in individual user profiles can reveal the structure of collective identity online, we perform a case study for a subset of users with the highest follower counts in our data. Due to their online visibility, these users are more likely to shape collective self-labeling behavior. We create a hashtag co-use graph of the top 2500 most-followed individuals and isolate its giant component, shown in Fig. 1a (1072 vertices, 12,178 edges, density=0.02). We use vertex color to differentiate clusters, which are annotated semantically using their top-4 ranked prototypical identity hashtags. Of note in this visualization is the separation of clusters based on political affiliation (*#maga/#kag* and *#resist/#theresistance*) versus those based on careers (*#producer/#actor*), and interests (*#travel*, *#instagram*, *#vegan*, *#fetish*, *#tech*, *#blockchain*). In Fig. 1b we illustrate how prototypical self-labels arise naturally from the edge density within the exemplar (*#author/#vegan/#poet/#fetish*) cluster. Edge colors here represent the presence, but not necessarily complete composition, of prototypical hashtags within edges. For example, pink edges indicate the presence of *#poet* in the absence of any of *#author*, *#vegan*, or *#fetish*, as exemplified in the {*#poet*, *#writer*} edge annotation. This entire cluster breaks down neatly into edge collections for each of the 4 prototypical tags, with some within-edge combination of these prototypical tags indicated with black edges (example edge annotated with {*#author*, *#poet*, *#amwriting*}, combining *#author* and *#poet*). Figure 1b thus provides visual evidence of how the top-n ranked hashtags by modularity score tend to concentrate in the highest-weighted and densest edges in in this cluster, as opposed to the example of the more peripheral {*#lesbian*} edge. These hashtags can be considered prototypical because of their importance in binding this cluster together (see “Methods” section for full description).

From the small-sample graph visualization in Fig. 1 we see how more specific semantic self-labels, not necessarily connected through their semantics (*#author* vs. *#fetish*), can still be associated through the clustering of individuals with similar self-concepts. This hints at a hierarchical structure emerging from self-labeling behavior, where a more specific concept integrates into a broader structure of affiliated concepts in the context of self-professed identity: for instance, the sexuality of *#fetish* and *#lesbian* labels resides inside the entire (*#author/#vegan/#poet/#fetish*) cluster, which may espouse the emergent identity of sexuality and writing.

## Hierarchy in the broad identity ecosystem

**Figure 2:**
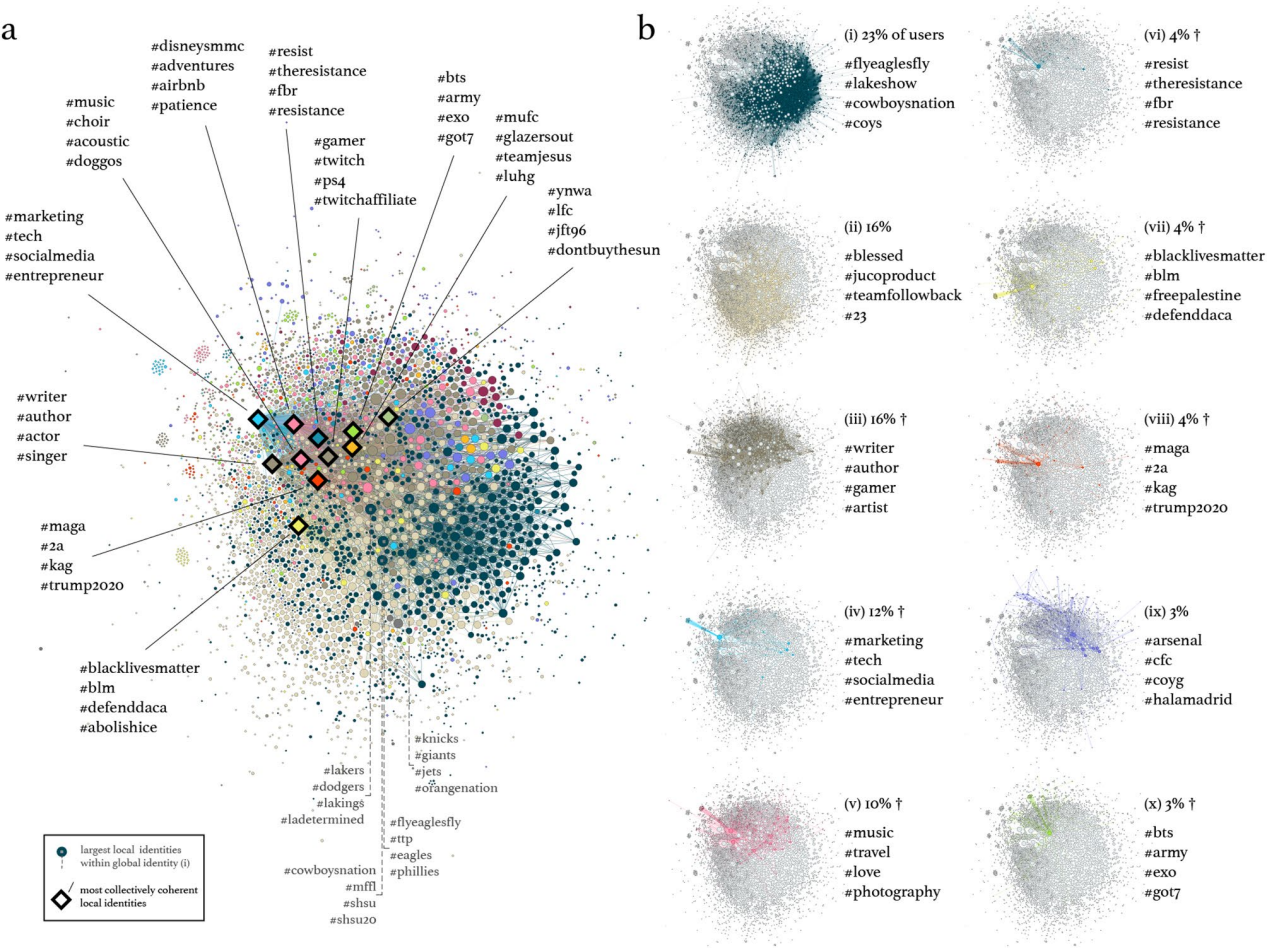
Visualization of collective identity label-sharing graph for over 85,000 users. Vertices represent local (bottom-level) clusters in a Louvain-derived cluster hierarchy; color represents global (top-level) cluster. (**a**) Total visualization, with the most collectively coherent identities labeled with diamond markers and annotated, as well as an illustrative labeling of the largest local clusters within the global sports cluster (i) in (**b**). (**b**) Individual visualizations of the top 10 largest global clusters with user coverage percentages. † Indicates high collective coherence with the corresponding local cluster marked with a diamond in (**a**). We observe marked difference in the coherence of local clusters with respect to their global status. The “tech professional” global cluster (iv) semantically matches and is dominated by its lone local counterpart in (**a**); a coherent identity from local to global levels of the hierarchy. Other global clusters, such as (i–iii), are diffuse in comparison, a difference we measure using information-theoretic methods.

We investigate how the self-labels of individuals collect into a hierarchical structure of online identity. The Louvain algorithm^16^ is a widely-used graph clustering algorithm that leverages modularity in stages, producing a hierarchical structure where local clusters aggregate in successive steps to a “global”, top-level partition. In this work we quantify the qualities of this hierarchy, relating local clusters at the bottom of the hierarchy $C_j^l$ to the top-level, global clusters which contain them $C_i^g$ (see SI Fig. 6). To visualize the entire identity graph spanning 85,839 users, we present the local clusters $C_j^l$ as vertices in Fig. 2, summing edge weights between users in any two clusters. To eliminate clutter in the visualization, we display only edges within a backbone of the graph^17^ (see SI Fig. 3 for the full graph). We characterize global clusters using color and characterize clusters by their prototypical hashtags as before. Fig. 2a profiles a selection of important and exemplar clusters semantically at the local level, while Fig. 2b pulls out the 10 largest global clusters to be visualized individually along with their own semantic annotations at the global level.

We find 4086 local clusters, collected into 18 global ones. A quick visual inspection shows the dominance in coverage by some global clusters, indicated by color. Sports fandom is a clearly dominant global identity, covering 23% of users in dark blue and characterized in cluster (i) of Fig. 2b. Its semantic annotation covers American and British sports teams (the latter referred to by *#coys*, “come on you spurs”). However, the Louvain-derived hierarchy captures more details of how identity collectives assemble. The largest 4 *local* clusters within the sports cluster (i), labeled in Fig. 2a, indicate the strong effect of regionalism on these sports fans: in ranked order of local cluster user size, these identities are Philadelphia, Los Angeles, Texas, then New York (state) sports fans (see SI for a glossary of hashtags).

The local cluster composition of each global cluster varies widely. At one end of a spectrum lie global clusters with high user coverage diffused over a wide base of their local clusters, like clusters (i–iii) in Fig. 2b and exemplified by the global sports cluster (i) already discussed. On the other end of the spectrum we find highly concentrated clusters like global clusters (iv, vi–viii, or x) (Fig. 2b), with only one or two obvious clusters dominating the local level. All 10 global clusters shown lie on this conceptual spectrum, i.e. the degree to which local identity clusters are more or less integrated or diffused with respect to this hierarchical composition.

## Characterizing the strength of collective identities

One consequence of aligning with an identity is that the individual becomes identifiable in the landscape of collective identity. We approach the effects of self-definition on “identifiability” in two ways, both of general interest for a user establishing identity online. The first focuses on the coherence of any particular collective identity: is my particular local identity only one facet of a broad identity type, or are my local and global identities nearly indistinguishable? The second focuses on the very act of self-labeling: if I use the self-labels of my particular local identity, how conspicuous am I among the sea of identifiers? To unify our treatment of these questions, we take an information-theoretic approach, as has been used to measure the divergence of language use in recent works^7,18–20^. For robustness, we repeat the following analyses using a different clustering technique, hierarchical stochastic block modeling (SBM)^21–25^, in SI Sect. 3.

### Coherence

From Figs. 1 and 2, we see evidence that encompassing identity clusters can assemble from more semantically specific, constituent identities. The sports cluster (i) in Fig. 2 exemplifies this, incorporating local fandom into a wide cluster of sports enthusiasts. But, how coherent is any global identity as a whole? Is being a Texas sports fanatic indicative of being a sports fan in general, at a global level? Given our peek into the diversity of local, regional sports clusters in global cluster (i), the answer would seem to be no. However, it seems that rabidly consuming K-pop in a local sense *does* seem to reflect global K-pop fandom (global cluster (viii) in Fig. 2, compared to its local counterpart). In other words, the collective K-pop identity (global cluster (x)) seems to be more coherent between its local and global levels than the collective sports identity.

And therefore, binary knowledge of a user’s membership in that *local* K-pop identity—whether they are or are not members—drastically reduces the uncertainty we have on their *global* identity membership. We call this uncertainty reduction for a given cluster its collective coherence, measuring it with the mutual information between binary local identity membership $Z^l_k$ and global identity membership $C^g$: $\mathrm {MI}\left( Z^l_k, C^g\right)$, where $Z^l_k$ distinguishes between membership in local identity $C^l_k$ versus all others $C^l_{\lnot k} = \bigcup _{j\ne k}C^l_j$, $C^g$ is the categorical distribution of global identities weighted by their user fraction, and *k* is the index of the local identity. When compared to the mutual information measured similarly for all other local identities, we create a comparative measure of identity coherence for each first-level, local identity shown in our map (Fig. 2a). We highlight the top 11 most collectively coherent local identities in this visualization with diamond markers, taken from the pronounced tail in the collective coherence distribution (see SI Fig. 5); their global counterpart identities are marked with †. We find similar coherence results when using an SBM clustering hierarchy, after taking the differing overall clustering behavior of the two models into account (see SI Sect. 3).

### Conspicuousness

Given the highly connected nature of our graph, local identities quite often share labels with other identities across identity boundaries. So although a collective identity may be coherent, its average member might not stand out from the general population as belonging to that specific identity. Users from such an identity would be highly conspicuous due to the identity’s typical vocabulary diverging from all others. This conspicuousness against the throng is readily measured using information-theoretic techniques. In this case, we measure how easy it is to determine the binary distinction $Z^l_k$ between local cluster $C^l_k$ versus the others $C^l_{\lnot k} = \bigcup _{j\ne k}C^l_j$ after observing a single label from the cluster’s vocabulary probability mass function *V*, embodied in the mutual information $\mathrm {MI}(Z^l_k, V)$. This is equivalent to the Jensen-Shannon divergence of *V* vs. the same for vocabulary of all local identities not $C^l_k$, a measure used to establish “distinguishability” between different vocabulary usages^18,20,26^ (see “Methods” section). This measurement can be made similarly for every local identity, creating a comparative measure of identity conspicuousness.

**Figure 3:**
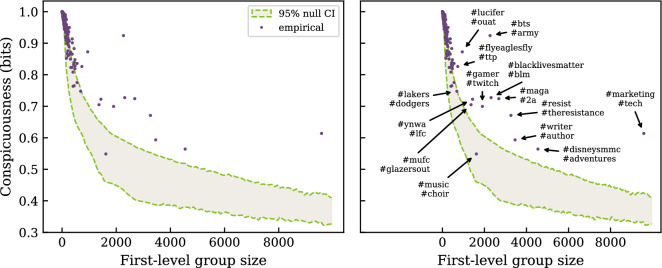
Conspicuousness vs. size for local identities. Conspicuousness measures the ease of recognizing a user’s identity group given its vocabulary. Conspicuousness generally decreases with increasing group size, pointing to an increased tendency for larger groups to use vocabulary shared in common over multiple groups. A simulated null model preserving users’ predilection for self-labeling, but randomizing specific label selection, shows a similar tendency without explaining conspicuousness levels for larger groups. This discrepancy points to additional processes in collective self-labeling which could create these more conspicuous identity vocabularies. Identities with over 600 users are annotated with the 2 self-labels contributing the most to the clustering of the identity.

Figure 3 plots these conspicuousness measurements for every local identity, versus their size (number of users; see SI for distribution of local cluster size). Conspicuousness for small clusters approaches 1, its upper limit, which makes sense given the construction of the graph; if these clusters shared more vocabulary with other users, they would likely be lumped into another local cluster by the resulting sharing of edges. Overall the trend to less conspicuousness with size points to a susceptibility of larger clusters to share more vocabulary with others. Of note, however, some clusters can buck the trend, such as the K-pop cluster labeled in Fig. 3 (*#bts*, *#army*).

We establish a base of comparison for these conspicuousness measurements with a null model preserving user self-labeling behavior but destroying the specific, empirical label selections each user makes. Behavior here refers to (a) any user’s predilection for self-labeling, as well as (b) any bias toward using more popular labels. We separate users into cohorts by how many self-labels they accrue, each with the associated pool of empirical labels used. For each cohort, we randomize the labels from its pool across its users, preserving the label count per user. Once all cohorts are randomized we construct the graph of label co-use over all cohorts as for the empirical data, finding its hierarchical cluster structure and measuring the conspicuousness of all local clusters. Figure 3 shows the 95% confidence interval band over cluster size from 31,190 null simulations. CI values represent the 2.5 and 97.5 percentiles of conspicuousness distributions per group size bin interval of 100: [1, 100], [101, 200], etc. 31,190 simulations guaranteed over 100 conspicuousness values generated per bin (see SI Fig. 7). This behavior-preserving null largely captures empirical conspicuousness at low cluster size, but does not explain that of most larger identities. See SI Sect. 3 for comparable results from hierarchical SBM clustering. This discrepancy hints at additional processes in collective self-labeling which could produce these more conspicuous vocabularies not created by this null.

## Discussion

Our chosen model of collective identity, leveraging simple graph structure and concentration of common self-labels into unique clusters per user, shows remarkable semantic results when considering the prototypical hashtags per cluster. On the other hand, each cluster shares many labels with others across the cluster landscape. This is one indication of how multifaceted a user can be in affiliation to different identity groups. Although placed in a primary cluster with a hard boundary, individuals in aggregate share many self-labels with other individuals in their own separate, primary clusters. Further work in mapping the structure of self-labeling in identity could include overlapping clusters.

As for the characterization of the collective identities in this work, it is interesting to consider what our two measures mean. First: we consider coherence. The procedure for creating the binary variable $Z^l_k$ for a particular local identity and then measuring coherence $\mathrm {MI}\left( Z^l_k, C^g\right)$ is an expression for how broad that identity is. In essence, it is how much we can *instantly* know about how diverse a user’s overall chosen identity is, integrating from local to global levels, in the landscape of identities. All of the annotated identities in Fig. 2a are highly coherent with respect to their global counterparts in Fig. 2b, distinct in coherence from the rest of the cluster population (SI Fig. 5). It is worth noting the presence of highly coherent identities corresponding to significant political movements of the time of data acquisition: *#maga*, *#resist*, and *#blacklivesmatter*. Especially in the context of the binary political environment associated with the movements, one might expect *#blm* and *#resist* identities to coalesce, but here both are coherent identities unto themselves. Other broad categories of these highly coherent identities could be considered lifestyle- or interest-driven (*#gamer*/*#twitch*, *#disneysmmc*/*#adventures*, or *#music*/*#choir* in Fig. 2a), vocation- or aspiration-driven (*#writer*/*#author*, *#marketing*/*#tech*), or associated with notoriously avid fandoms (*#bts*/*#army*, *#mufc*/*#glazersout*, and *#ynwa*/*#lfc*—the latter two identities relate to different football clubs, see SI Sect. 4).

Second, we consider conspicuousness $\mathrm {MI}(Z^l_k, V)$: the average ease an observer has identifying a particular identity source vs. all other identities given a single self-label^18^. To capture a specific scenario of a newcomer to online identities, we make this newcomer naive to the probability of encountering a self-label from any specific identity (see “Methods” section). The divergence of empirical conspicuousness against our null simulations is quite interesting—here the location outside the 95% CI band is not indicative of any kind of “significance” of the measurement. Instead, it indicates a difference in outcome from what could be expected given the behavioral characteristics preserved in the null model. In this case, preserving user characteristics of quantity and choice for niche vs. widespread self-labels does not produce the greater levels of conspicuousness observed. Further work could involve the addition of other dynamics involving choice of self-labels, such as diachronic processes involving the assembly of self-labels for users establishing public identities.

The connection to Social Identity Theory (and specific subset Self-categorization Theory (SCT)) of this work is loose, focusing on elements of our system of study that could plausibly represent elements of these theories. Briefly, in SCT social groups are maintained by two actions of member individuals: (i) increasing personal characteristics similar to other members in-group, and (ii) decreasing personal characteristics similar to individuals outside their group^10^, a known trait of human psychology^27^. In this work, self-labeling via hashtags could be thought of as a mechanism for the former, increasing in-group similarity of identity. We empirically define identity as the emergence of common patterns of self-labeling, found via graph clustering. We also use linguistic tokens as actions in group identity maintenance, a perspective more common in earlier manifestations of SCT; although recent calls have been made to reintroduce language-based practices into SCT^10^.

## Conclusion

In this work, we collect an unprecedented data set of self-labeling on Twitter, harvesting labels inherently connected to a user’s sense of identity from their presence in designated self-descriptions. We take a straightforward approach with an empirical mapping of collective identity via label co-use and unsupervised identity alignment using modularity-maximization. The hierarchical identity structure we obtain exposes variation in how users collect into identities through the process of self-labeling: our coherence measure documents a spectrum of collective identity behavior. We find that some collective identities are more collectively coherent, including the notable examples of political movements during the time period. In a parallel analysis, we measure how conspicuous each collective identity is compared to the overall identity landscape. The conspicuousness of larger communities is not explained by a behavior-preserving null model of self-labeling. We obtain similar results for coherence and conspicuousness with an analogous analysis using hierarchical stochastic block modeling (see SI). An interesting followup study would investigate diachronic processes in self-labeling behavior that could produce the empirical conspicuousness seen in this work.

## Methods

Our research project does not involve human participants. We collected the relevant Twitter data without subject interaction, using only data that already existed and that was collected for a reason other than use in this research study. Our data collection and analysis has been reviewed and deemed “Exempt” by the Indiana University Institutional Review Board (Protocol #1707249405). Our Twitter data is furthermore de-identified by the removal of all individual identity markers, stored on a secure server, and subsequently analyzed in the aggregate without references to the identities of individual Twitter users.

All tweet records harvested for this work come from the Twitter streaming “decahose”, a quasi-random sample of 10%^28^ of daily tweets, made available specifically to researchers at Indiana University through the decahose archive of the Observatory on Social Media (OSOME)^12^. The user profiles are held in the “user” object within each tweet’s JSON record provided, under the “description” field. Bots are removed from this sample using Botometer^2,29^, a service which examines an account’s content and other meta-data arriving at an accurate estimation of its probability of being a bot or a person. After consultation with the creators of Botometer, standard practice is to classify any user with a score $>\,0.5$ as a bot (score ranges from 0 to 1). To increase our accuracy, we consider any user with an English bot score $>\,0.4$ to be a bot.

Of great importance is the difference between the timing of the tweet record and the timing of the profile description provided within the tweet record. Specifically, the tweet provides exact timing information of the tweet itself, but only an upper bound for the timing of profile description creation. Any user can alter their profile description at any time, and the change is not represented in API-derived data until the next time that user tweets and the tweet is provided in the streaming sample. We are interested in a cohort of users who actively maintain their profiles. Therefore, we sort all tweets harvested for a user chronologically, then retain only tweet objects where the profile changes from the one harvested before (via difference of token sets). This necessarily excludes the earliest profile description harvested for any user, and establishes that all subsequent profiles were edited and therefore actively maintained in the given period of time.

We apply the following methodology to create our user sample: we pre-screen existing decahose samples from 2012, 2013, and 2014 for users exhibiting active profile maintenance, defined as at least one profile edit within each year, producing an initial user pool of 10,737,093. Using Botometer scores we exclude users with bot scores higher than the mentioned conservative threshold (based on their latest 3200 tweets up to Fall 2019), producing a potential user pool of 4,755,274. We then harvest the decahose tweets of these same users occurring from August 2017 through December 2019, creating 2,089,173 user profile change timelines derived from their retrieved tweets. Of these, 1,972,863 have only English as their specified language in their user metadata. 347,252 of these users have profiles containing hashtags, and 121,084 have timelines certain to lie in 2017-08-01 through 2019-12-31 after discarding their initial profile description (described above). Furthermore, we exclude organization accounts marked by the Humanizr classifier^30^, retaining 113,034 user accounts that were not deemed organizational but belonging to individuals, then create a graph with the remaining 91,093 users who share at least one hashtag with another user. The final cohort contains the 85,839 users contained in the giant component of this graph and retained after removing those sharing hashtags with only a single other user.

We create a graph model *G*, with the vertex set denoting users and an adjacency matrix *A* representing edge weights between users (see SI for graph metrics including degree distribution). The edge weight $A_{ij}$ for any connected pair of users (*i*, *j*) is the number of identical hashtags that their profiles both contain. In addition, we recover which hashtags contribute to each edge weight via the function $T:(i, j) \mapsto \left\{ \phi :\phi \in \Phi \right\}$, mapping edges to their associated subsets of hashtags within encompassing tag superset $\Phi$. To maximize alignment in shared identity held in the edge weights of *G*, we partition the graph via modularity-maximization because of its focus on clusters with high edge concentration. Given edge weight $A_{ij}$ the degree of vertex *i* is $k_i = \sum _j A_{ij}$ and modularity per edge $B_{ij} = A_{ij} - \frac{k_i k_j}{2m}$, where $m=\sum _{ij}A_{ij}$. Using partition variable $c_i$ to label vertex *i* with its cluster membership and Kronecker $\delta _{ij} = 1$ if $c_i = c_j$ and 0 otherwise, total modularity of graph *Q* can be written^15^:

1 $$\begin{aligned} Q(B, c)&= \frac{1}{4m} \sum _{ij}B_{ij} \delta _{c_i,c_j} - \frac{1}{4m} \sum _{ij}B_{ij} (1 - \delta _{c_i,c_j}) \nonumber \\&= \sum _{ij}q_{within}(i, j, c) - \sum _{ij}q_{between}(i, j, c). \end{aligned}$$

Because $\delta _{ij} = 1$ only when both vertices *i* and *j* are within the same cluster, the formulation of Eq. (1) emphasizes the positive contribution to modularity for edges (*i*, *j*) *within* clusters vs. the negative contribution of edges *between* them.

This is an expression of the accumulation of modularity, or identity alignment, over each and every edge, given a partition *c*. But, edges are comprised of identity labels, so we can re-frame the sum as an accumulation of modularity over the identity labels present proportionally in each edge. In particular, we can constrain the sum and consider the identity alignment contributed by only a specific label if present in any edge. We define this alignment contribution by label $\phi$ as

2 $$\begin{aligned} K(\phi , B, c)&= \sum _{ij}T^\phi _{ij}q_{within}(i, j, c) - \sum _{ij}T^\phi _{ij}q_{between}(i, j, c), \end{aligned}$$

where $T^\phi _{ij}$ is the fraction of weight given to edge (*i*, *j*) by $\phi$: $T^\phi _{ij} = |T(i, j) \cap \{\phi \}|/|T(i, j)|$. Given a partition *c* into clusters that maximizes modularity *Q*, we can find how much each hashtag $\phi$ contributes to *Q* *of the entire graph* using *K*. We can also focus on a specific cluster, considering only edges with at least one vertex belonging to that cluster. The hashtags with highest $K_c$ calculated using only this limited edge set are the ones most important to identity alignment *of the cluster of interest*. The prototypical hashtags used to annotate specific clusters throughout this work are those with highest $K_c$ for the community *c* being described. Force-directed layout of *G* and its presentation were produced via Gephi software. We prune user vertices with degree 1 in the full user-user network before applying the Louvain algorithm. The multiscale backbone was calculated using a significance threshold $\alpha =0.001$^17^.

In the main text we allude to the equivalence of $\mathrm {MI}(Z^l_k, V)$ and Jensen–Shannon divergence, which we show here. We have binary variable $Z^l_k$ distinguishing outcomes from cluster *k* or not. In general, we can take a binary $Z \sim \mathrm {Bernoulli}(\alpha )$, with outcomes $\{z_0, z_1\}$. We consider the probability of label *v* given one outcome of *Z* as $p(V=v | Z=z_0) = p_0(v)$, written similarly for $p(V=v | Z = z_1)$, with total probability for $v \in V$ as $p(v) = \alpha p_0(v) + (1-\alpha )p_1(v)$. Mutual information $\mathrm {MI}(Z, V) = \mathrm {H}(Z) - \mathrm {H}(Z|V)$, where *H* refers to the Shannon entropy. Starting with the second term, $\mathrm {H}(Z|V) = -\sum _{z,v}p(z,v)\mathrm {log}_2p(z|v)$ and using identity $p(z|v) = p(v|z)p(z)/p(v)$:

$$\begin{aligned} \mathrm {H}(Z\vert V) =&-\sum _{z,v}p(v|z)p(z)\mathrm {log}_2 \left( \frac{p(v|z)p(z)}{p(v)} \right) \\ =&-\alpha \sum _v p_0(v) \mathrm {log}_2 \left( \alpha \frac{p_0(v)}{p(v)} \right) \\&-(1 - \alpha ) \sum _v p_1(v) \mathrm {log}_2 \left( (1 - \alpha ) \frac{p_1(v)}{p(v)} \right) \\ =&-\alpha \mathrm {log}_2(\alpha ) -(1 - \alpha ) \mathrm {log}_2(1 - \alpha ) \\&-\alpha \sum _v p_0(v) \mathrm {log}_2 \left( \frac{p_0(v)}{p(v)} \right) - (1 - \alpha ) \sum _v p_1(v) \mathrm {log}_2 \left( \frac{p_1(v)}{p(v)} \right). \end{aligned}$$

The first two terms represent $\mathrm {H}(Z)$, so the original mutual information is equivalent to the Jensen–Shannon Divergence (JSD):

$$\begin{aligned} \mathrm {MI}(Z,V) = \mathrm {JSD}(p_0, p_1, \alpha ) = \alpha \mathrm {KL}(p_0 | p) + (1-\alpha ) \mathrm {KL}(p_1 | p), \end{aligned}$$

where $\mathrm {KL}(p|q)$ is the relative entropy of probability mass functions *p* & *q*: $\sum _v p(v) \mathrm {log}_2 \left( p(v)/q(v) \right)$. Specifically for this case, the JSD is the expected relative entropy with respect to the total vocabulary distribution over both cluster and non-cluster sources weighted by the probability of observing the cluster itself, $\alpha$. We elect to embody an *unbiased* observation approaching this identity landscape with no knowledge of how prominent any identity cluster is. Then, $Z^l_k$ is uniform, i.e. $\alpha = \frac{1}{2}$^18,20,26,31^.

## Supplementary Information

Supplementary Information.

## Data Availability

The code and datasets generated and/or analysed during the current study are available in the GitHub repository https://github.com/CogentMentat/Quantifying_Collective_Identity_from_Self-defining_Hashtags_Data_Code, archived at Zenodo repository 10.5281/zenodo.6942603, in the following forms: Per the Twitter Developer Agreement and Policy, raw tweet and profile data is not allowed to be redistributed, but we do include a supplementary file of the 91,093 Twitter User IDs of the graph analyzed in this work. Profile data for these users may be harvested through the Twitter API. We also provide the edge list of the same graph, with hashtags per edge, without User ID information.
