# Supplementary material for: Quantifying collective identity online from self-defining hashtags
Source: Sci Rep. 2022 Sep 3;12:15044. doi: 10.1038/s41598-022-19181-w (PMC9440909; doi:10.1038/s41598-022-19181-w)
Supplement: Supplementary file 1 — Supplementary Information. [file 41598_2022_19181_MOESM1_ESM.pdf]

Quantifying online identity from self-defining hashtags: Supplementary Information

Alexander T. J. Barron*, Johan Bollen

Contents

1 Graph metrics **2**

2 Louvain clustering supplementary information **5**

3 Complementary results using hierarchical Stochastic Block Modeling **9**

3.1 Coherence using an SBM hierarchical partition 9

3.2 Conspicuousness at different SBM levels 17

4 Hashtag glossary for main text **18**

*: Corresponding author (atbarron@iu.edu)

1 Graph metrics

Our graphs consist of user nodes, where edges between users indicate the co-use of at least one hashtag with another user in self-description profiles. We begin with 91,093 user nodes, with largest connected component consisting of 89,647 nodes; component size distribution is shown in Table 1. We then perform all our analyses on the 2-core of the giant component, removing users that share hashtags with only one other user. Table 2 contains graph metrics for that 2-core. Its path length histogram is shown in Figure 1; degree distribution and associated heavy-tail fit information are shown in Figure 2 and Table 3.

Component size	Count
89,647	1
8	3
6	2
5	3
4	18
3	81
2	540

Table 1: Connected component size distribution for the original user identity graph.

vertex count	85,839
edge count	14,753,136
density	0.004
clustering coefficient	0.514
assortativity by vertex degree	0.284

Table 2: Graph metrics for the giant component 2-core, our graph of interest.

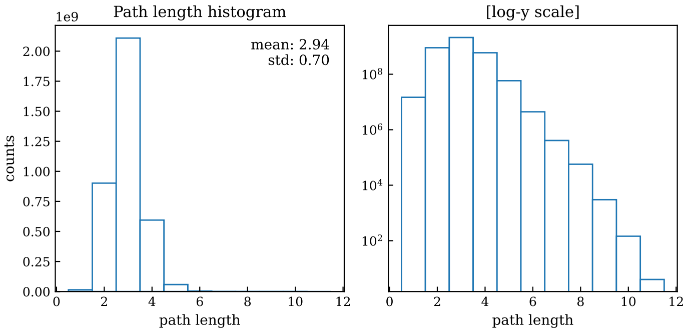

Figure 1: Histogram of path lengths for the graph 2-core.

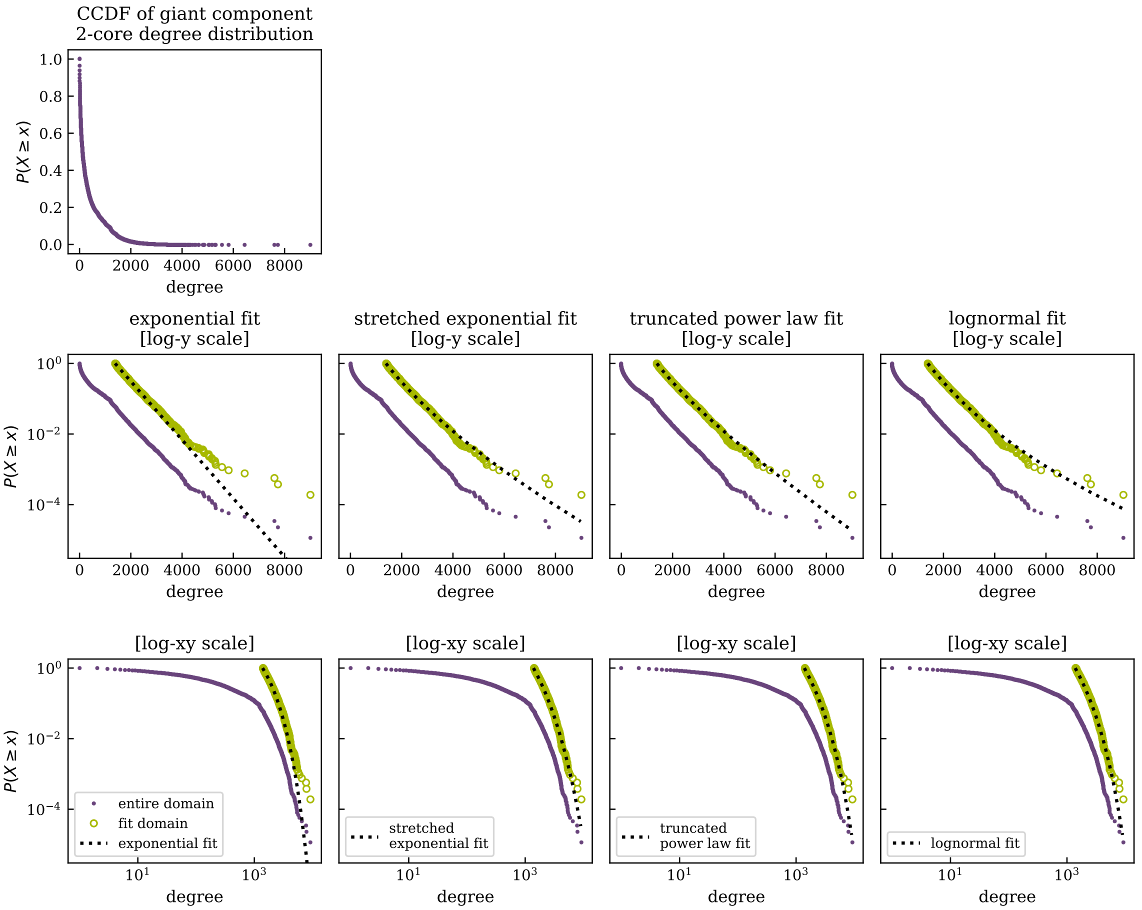

Figure 2: Complementary cumulative distribution of degree for the giant component 2-core identity graph: the graph used to obtain the clustering hierarchy visualized in Figure 2, main text. Comparison of different model fits for the heavy tail are shown. According to the methods of Clauset et al. and Klaus et al., using the tools developed by Alstott et al.[2, 3, 1], the heavy-tail behavior in the degree distribution is decidable between stretched exponential, truncated power law, and lognormal models (see Table 3 for log-likelihood comparison results).

(R, p)	power law	truncated power law	exponential	stretched exponential	lognormal
power law	–	–	–	–	–
truncated power law	(54.40, 0.000)	–	–	–	–
exponential	(27.87, 0.106)	(-26.53, 0.001)	–	–	–
stretched exponential	(53.55, 0.000)	(-0.85, 0.234)	(25.68, 0.000)	–	–
lognormal	(51.45, 0.000)	(-2.95, 0.082)	(23.58, 0.008)	(-2.10, 0.040)	–

Table 3: Log-likelihood fit comparisons for an array of heavy-tail models on the degree distribution shown in Figure 2. Each cell shows (R, p): R is the log-likelihood ratio of the *i*th row model fit against that of the *j*th column. Positive R indicates a preference for the *i*th row model, with negative indicating the *j*th column model. p indicates the p-value associated with each R as calculated in [1]. Truncated power law, stretched exponential, and lognormal are preferred over exponential and power law, although no preference is shown among the former three.

2 Louvain clustering supplementary information

Figure 3 shows the graph of 1st-level (local) clusters with all edges shown (instead of the backbone as in Fig. 2, main text). Figure 4 shows the size distribution of these first-level clusters and for the two higher levels in the Louvain hierarchy. Figure 5 shows the distribution of collective coherence measured, as well as the threshold used to label high-coherence clusters in Fig. 2a, main text. Figure 6 contains an explanatory diagram of hierarchical identity structure, and Figure 7 contains details of null conspicuousness sample sizes used to create the null conspicuousness CI band.

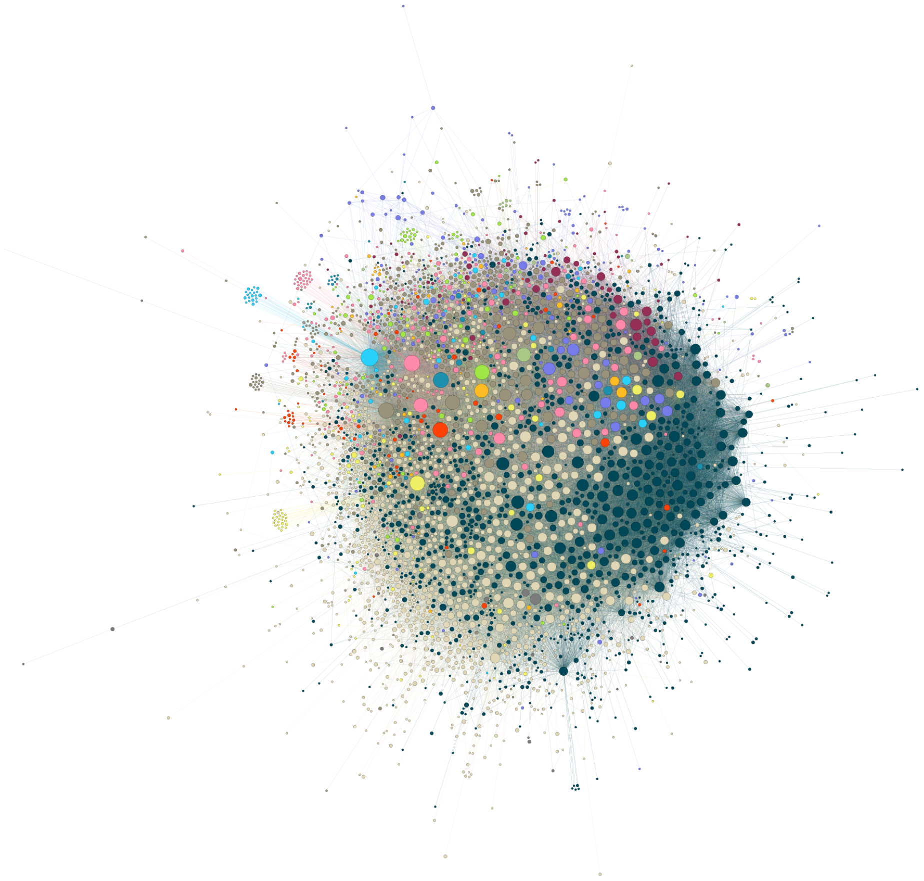

Figure 3: Complete force-directed visualization of 4,086 Louvain first-level communities, as in Fig. 2 main text, with all edges shown. The main text figure shows only edges in the multiscale backbone[9] with $p < 0.001$.

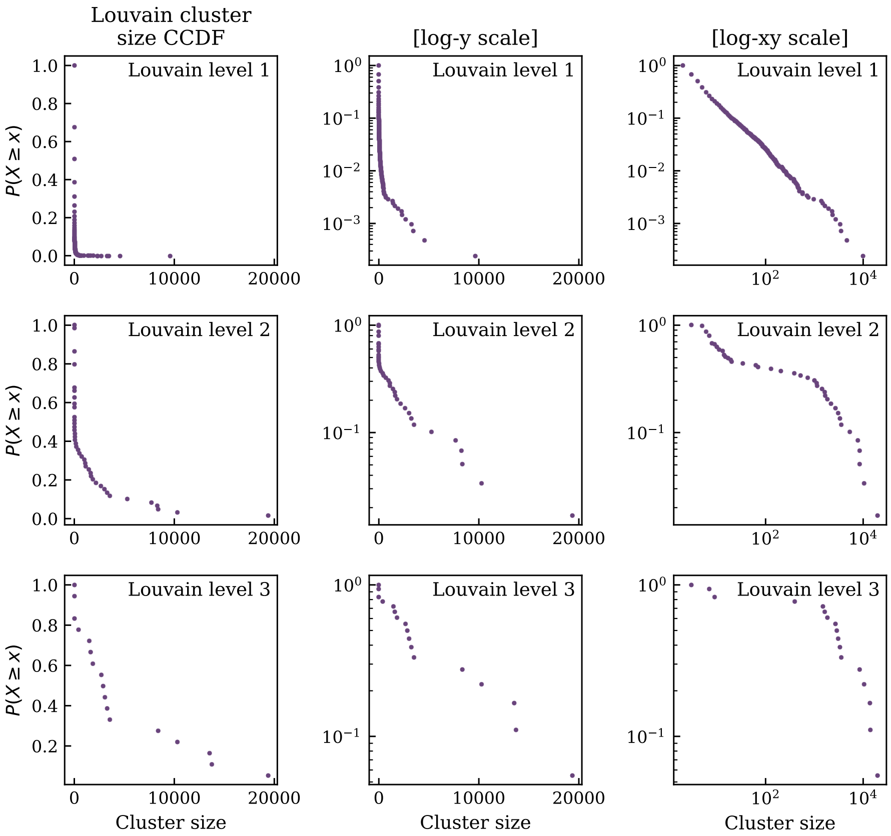

Figure 4: Cluster size distributions per Louvain level, shown in different combinations of logarithmically- and linearly-scaled axes.

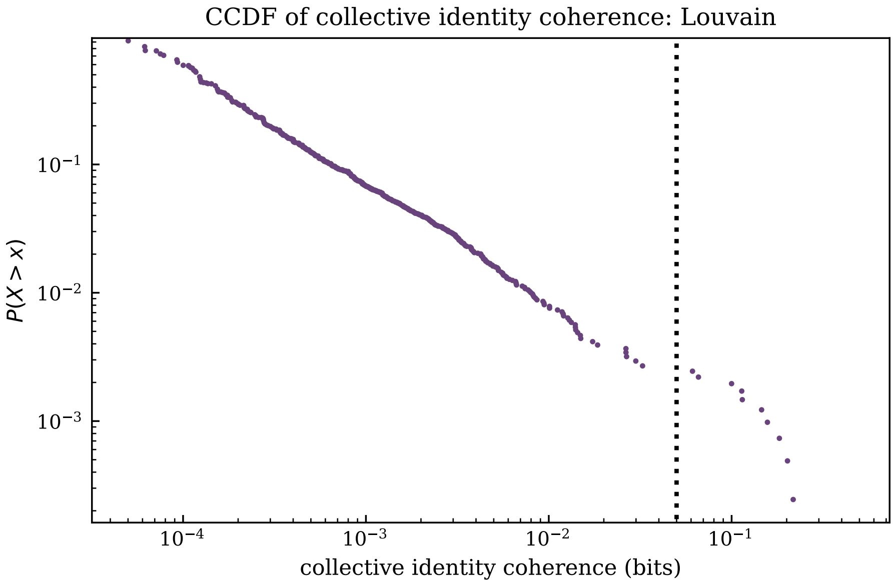

Figure 5: Complementary cumulative distribution of collective coherence of all local, first-level Louvain communities. Vertical line indicates the threshold used to select high coherence communities to label in Fig. 2a in the main text.

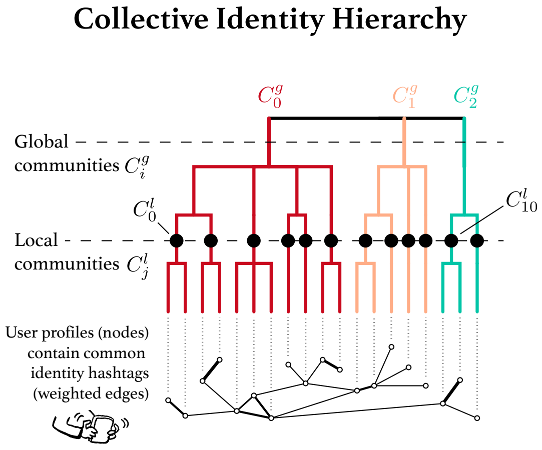

Figure 6: Explanatory diagram of hierarchical identity structure. User vertices connected by edges representing commonly-used self-labeling hashtags are collected together in stages to form the full collective identity hierarchy. Communities at the bottom, local level C_j^l aggregate into their top-level, global counterparts C_i^g.

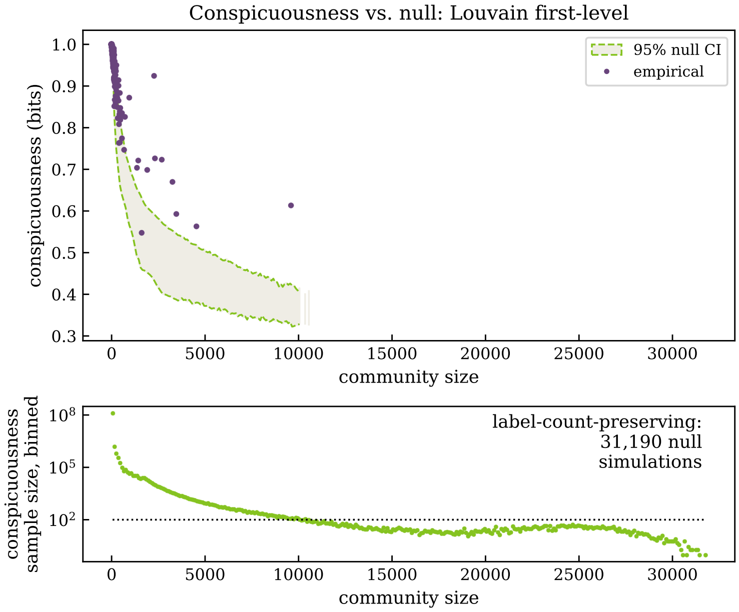

Figure 7: Top: empirical Louvain first-level conspicuousness results presented with the 95% confidence interval (CI) band of null conspicuousness values. CI values represent the 2.5 and 97.5 percentiles of conspicuousness distributions per group size bin interval of 100: [1, 100], [101, 200], etc. CI ranges are plotted for only those bins with more than 100 conspicuousness values. Bottom: generated conspicuousness sample size per null community size bin. The horizontal dashed line represents the threshold of 100 samples needed to plot the CI in the top plot corresponding to any bin. Using this threshold, more than 30,000 simulations were needed to produce the CI band covering the range of empirical community sizes (up to roughly 10,000 users).

3 Complementary results using hierarchical Stochastic Block Modeling

3.1 Coherence using an SBM hierarchical partition

In the main text, we produce a clustering hierarchy using the Louvain algorithm, and compare clusters from the bottom level against the top level to produce coherence scores. Our analysis is dependent on the Louvain algorithm to find clusters representing assortative communities of self-labelers, so to strengthen our findings we compare against coherence measurements from a hierarchical stochastic block model (SBM)[5, 6, 7]. All SBM calculation is performed with the graph-tool library[4], using a geometrically-distributed edge covariate model to represent positive integer edge weights (see [6]). We present results from the SBM model with the smallest description length (entropy) out of five attempts (59166727 vs. 59259313, 59320690, 59400638, and 59677231, truncated to natural numbers) [7].

The hierarchical SBM provides a rigorous inferential approach to complement the Louvain hierarchy. Although the SBM does not specifically infer partitions based on network assortativity, its implementation as described in [5, 6, 7], and implemented in the graph-tool library, makes minimal model assumptions and can infer clusterings that might stem from a number of different generative processes besides assortativity. We present statistics for both the Louvain and SBM clusterings by level in Tables 4 and 5; the former produces three levels, while the SBM finds 12. Figures 4 and 8 contain cluster size distributions per level for the Louvain- and SBM-based hierarchies.

Level	Modularity	Cluster count	Mean cluster size	Min cluster size	Max cluster size
1	0.582	4,086	21.01	2	9,582
2	0.623	59	1,454.90	3	19,316
3	0.624	18	4,768.83	3	19,337

Table 4: Table of clustering measurements by level for the Louvain hierarchy.

Much of our Louvain-based analysis makes use of modularity: both the definition of clusters themselves and the ranking of prototypical self-labels per cluster rely on modularity as a quantity. To compare the two clustering approaches, we calculate modularity for both at each level. As expected, the Louvain algorithm increases modularity of its partition at every level. The SBM, in contrast, seeks to minimize the minimum description length of its inferred nested partition, and therefore need not maximize modularity at any level[5]. It is noteworthy, then, that SBM-produced partitions increase in modularity with every level up through level 6 (Table 5).

Level	Modularity	Cluster count	Mean cluster size	Min cluster size	Max cluster size	1st-level Louvain overlap	Top-level Louvain overlap
1	0.124	1,764	48.66	1	650	0.297	0.044
2	0.180	818	104.94	1	1,056	0.277	0.091
3	0.298	464	185.00	1	3,685	0.286	0.188
4	0.372	240	357.66	1	10,269	0.244	0.256
5	0.456	167	514.01	1	18,287	0.211	0.347
6	0.465	85	1,009.87	1	33,035	0.181	0.369
7	0.457	46	1,866.07	1	56,510	0.158	0.353
8	0.022	17	5,049.35	1	78,911	0.114	0.239
9	0.022	6	14,306.50	30	78,911	0.113	0.236
10	0.021	3	28,613.00	118	78,911	0.108	0.234
11	0.021	2	42,919.50	6,928	78,911	0.108	0.234
12	0.000	1	85,839.00	85,839	85,839	0.112	0.225

Table 5: Table of clustering measurements by level for the SBM hierarchy.

Our original coherence measure compares the bottom level of the Louvain hierarchy against the top. To compare coherence results, we need to establish which SBM levels to use as upper and lower levels in a similar way. We calculate clustering similarity between our Louvain levels and every

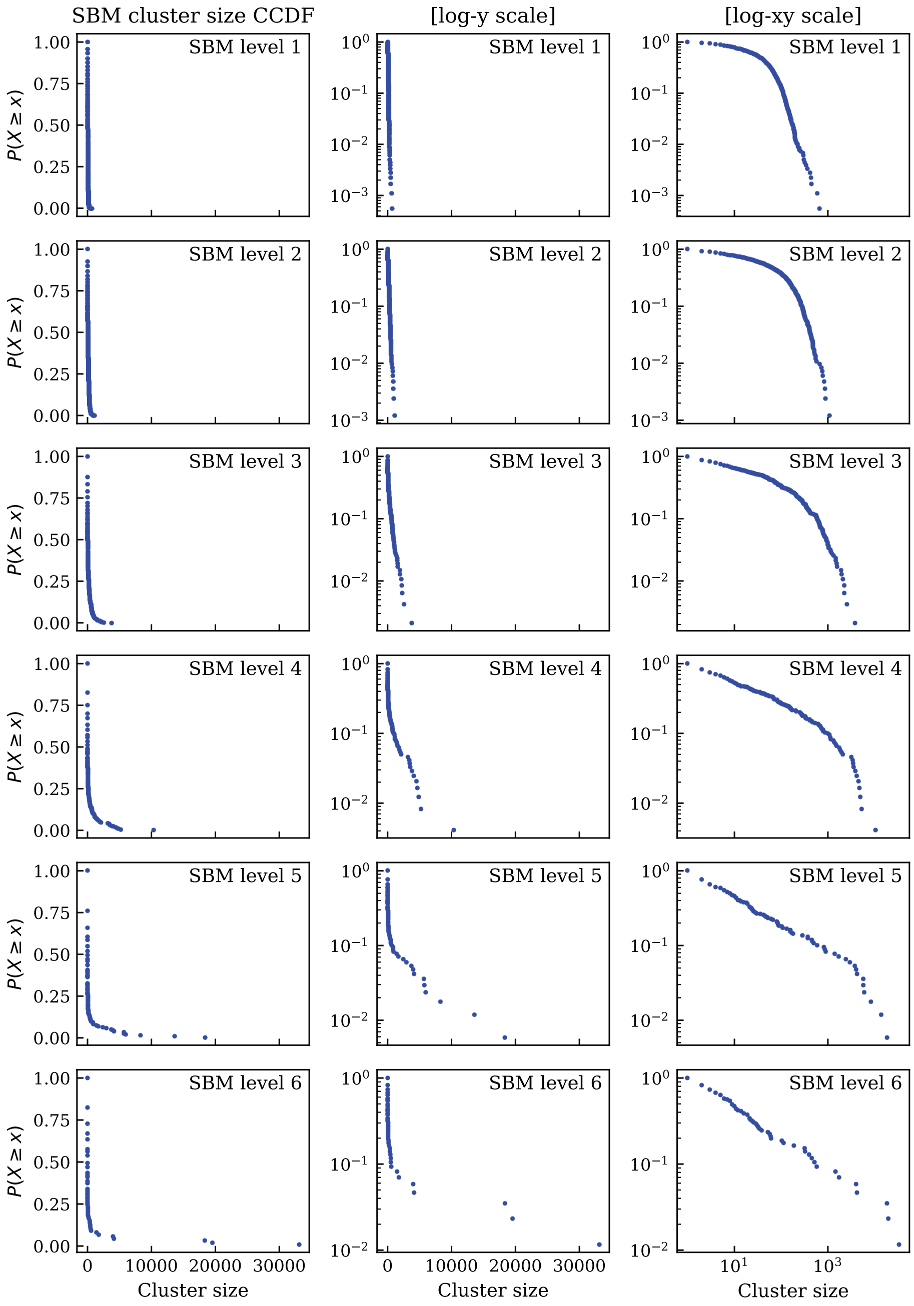

Figure 8: Cluster size distributions per SBM level, shown in different combinations of logarithmically- and linearly-scaled axes. We show up through level 5, the highest level relevant to our comparison analysis.

SBM level via their normed maximum partition overlap similarity score, provided by the graph-tool partition_overlap function[8, 4], presented in Table 5. We choose SBM level 6 as the upper level for calculating coherence as the level that achieves maximum modularity and maximum overlap with the Louvain top-level clustering.

We find that the hierarchical SBM produces a first-level cluster landscape that "breaks up" the larger, high-coherence clusters found in the first level produced by the Louvain algorithm (see Figure 9). However, the SBM coalesces the clusters containing the user vertices common to these high-coherence Louvain clusters, recovering them increasingly with rising level. In the main text, we highlight these highest-coherence bottom-level Louvain clusters. We provide evidence in Figure 10 that these particular high-coherence clusters are significantly recovered as SBM layer increases. We match each Louvain cluster to the singular SBM cluster in each level that shares the greatest overlap in user nodes, then plot the its coherence score for the Louvain-based clustering against the overlap with its SBM counterpart. As the SBM level increases, we see that the high-coherence clusters are exactly those which are recovered, shown by the increased overlap with matched, single SBM clusters. The log-x plots show that a population of the smallest, least coherent clusters are "found" by SBM by the first layer; it takes increasing layers to recover more of the larger, more coherent Louvain bottom-level clusters to the same degree.

Overall, it seems as though the hierarchical SBM produces more hierarchical levels, with the lower levels consisting of smaller and more uniformly-sized clusters (Table 4 vs. Table 5; Figure 9). This is not unexpected, as seen in comparisons between generative model-agnostic SBM clusterings vs. "planted partition" SBMs with assortativity-seeking generative models: the general SBM tended to break up the larger clusters found by the planted partition SBM[10]. Unfortunately there was no option to use the planted partition model hierarchically, as accomplished in [5] and [6], since the planted partition model has not been implemented in a hierarchical way in the graph-tool software as of May 2022.

We calculate coherence distributions for lower SBM levels with respect to SBM level 6, established earlier. Only by level 3 does the coherence distribution reach the original Louvain high-coherence range (Figure 11). Therefore, we use SBM levels 3 and 4 vs. 6 as our lower- vs. upper-level analogs for the Louvain bottom- and top-level clusterings presented in the main text. Figure 12 shows a direct positive relationship between SBM- and Louvain-derived coherence scores, where clusters between models are matched via overall cluster overlap maximization and implemented in scipy's linear_sum_assignment function.

Tables 6, 7 and 8 show the top-10 prototypical self-labels for high-coherence lower-level clusters in Louvain and SBM cases. In terms of the face-validity of labels describing each cluster, SBM recovers many of the original Louvain-dependent results, with some additional clusters above the 0.05 bit threshold. However, the point is not to recover clusterings exactly, but to show a consistent variation in the structure of the identity cluster hierarchies–one where particularly coherent communities provide more information about their encompassing higher-level identity landscapes than others. Both Louvain- and SBM-derived measurements establish this, with significant recovery of specific results.

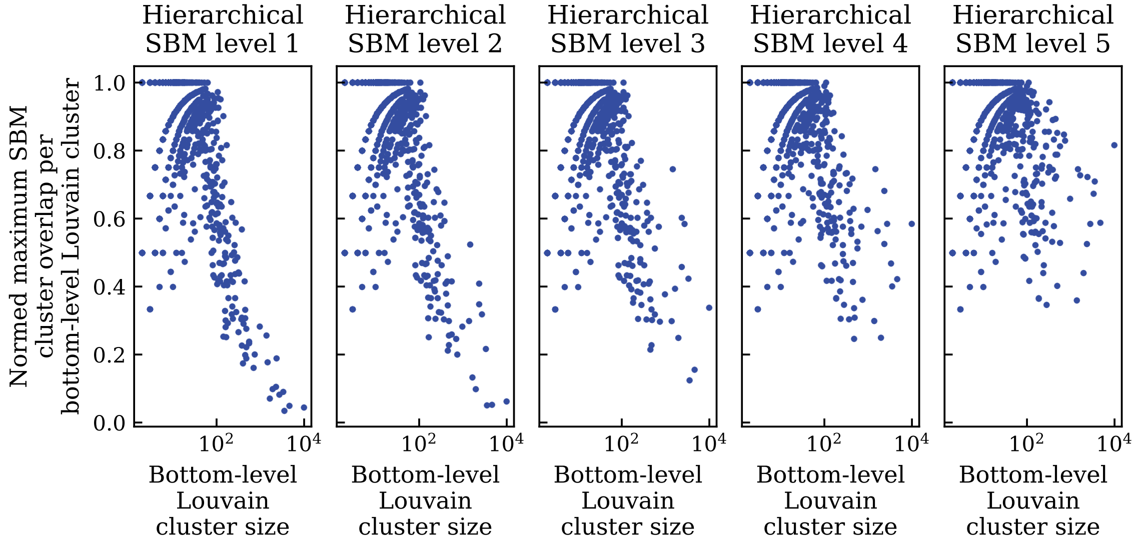

Figure 9: Overlap of SBM clusters matched by greatest overlap to each Louvain bottom-level cluster, plotted against Louvain cluster size on the x axis. Overlap is the matched clusters' intersection cardinality normalized by the Louvain cluster cardinality. Large Louvain clusters are "broken up" by the SBM at lower SBM level, but as the level rises, the largest Louvain clusters are recovered increasingly by the SBM.

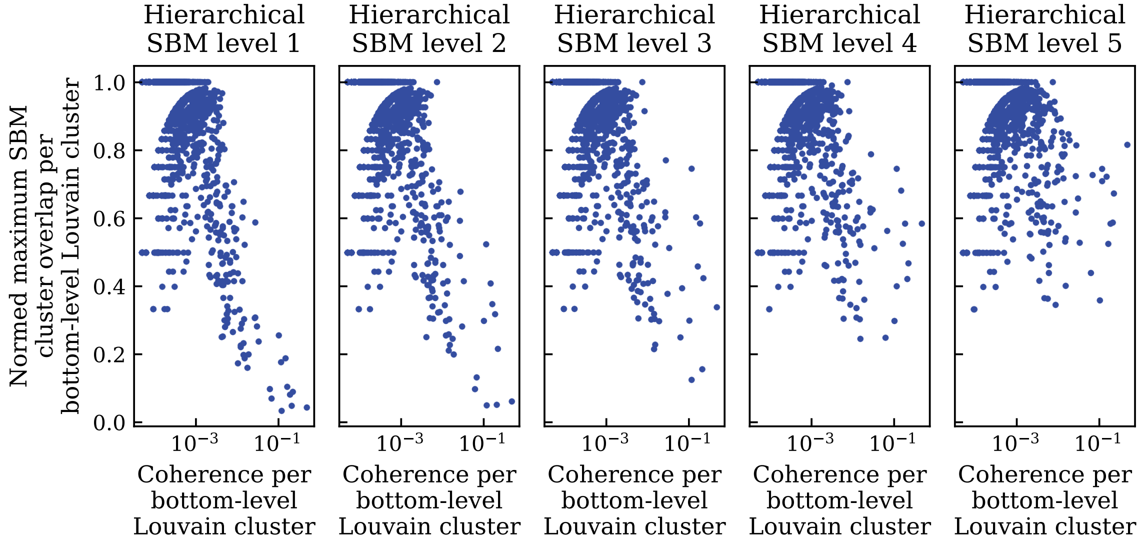

Figure 10: Overlap of SBM clusters for each Louvain bottom-level cluster as in Figure 9, but plotted against the Louvain clusters' coherence scores. The high-coherence Louvain bottom-level clusters defined in Figure 5 and shown in Figure 2, main text, tend to be recovered increasingly by the SBM with rising level. Compare Table 6 to Tables 7 and 7 to see recovery of the prototypical labels of high-coherence Louvain clusters from the SBM.

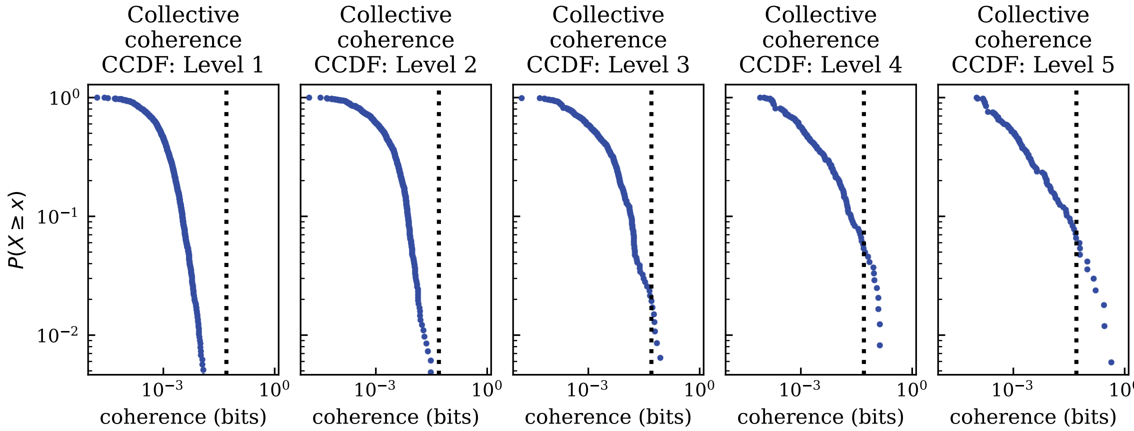

Figure 11: Coherence distributions for all clusters in each increasing SBM level, measured with respect to SBM level 6. Only by level 3 does the coherence distribution range reach that of the Louvain-derived coherence distribution, as shown by the common threshold line with Figure 5.

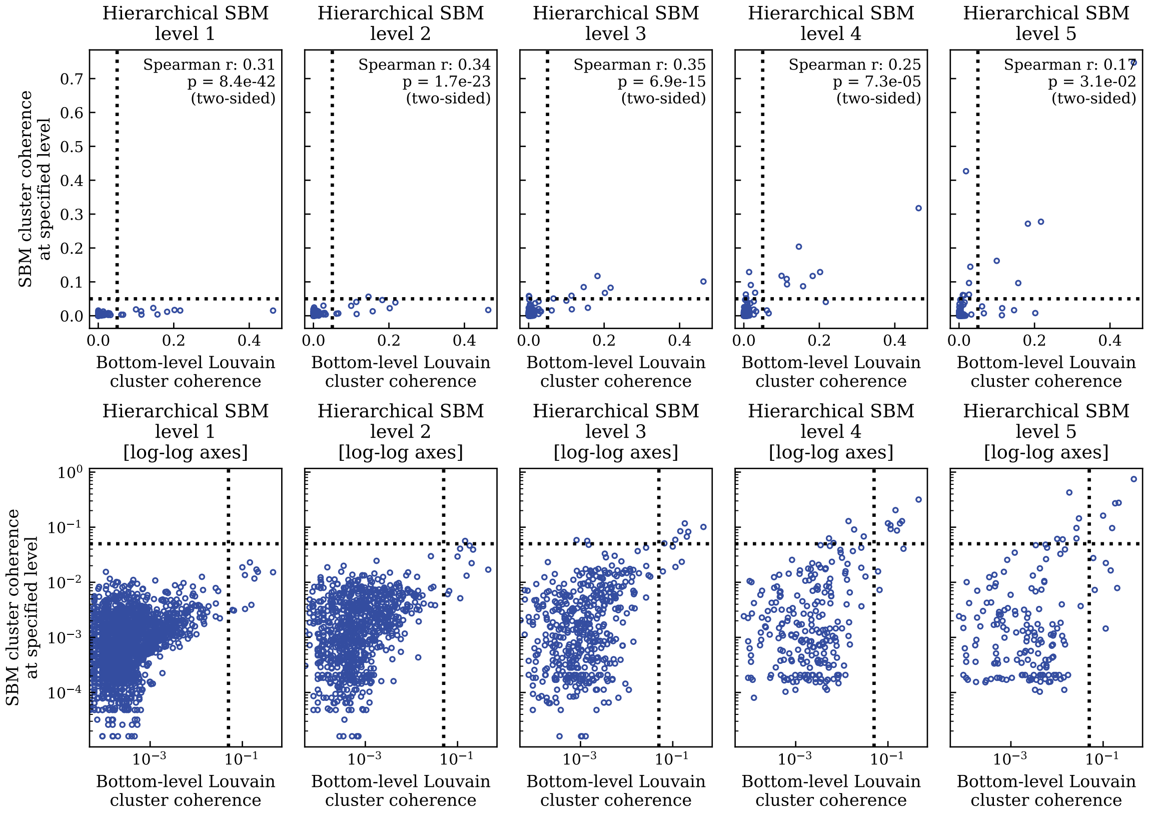

Figure 12: Scatter plots of SBM cluster coherence vs. Louvain cluster coherence, where clusters between models are matched by maximizing the sum of matched overlap values overall and implemented in scipy's linear_sum_assignment function. The the bottom row plots are log-log axes versions of the top row, and dotted lines indicate the same threshold as shown in Figures 5 and 11. We find a positive relationship between SBM and Louvain-derived coherence measurements.

Cluster a	Cluster b	Cluster c	Cluster d
#marketing	#resist	#disneysmmc	#maga
#tech	#theresistance	#adventures	#2a
#socialmedia	#fbr	#airbnb	#kag
#entrepreneur	#resistance	#patience	#trump2020
#ai	#bluewave	#histmed	#wwg1wga
#digital	#impeachtrump	#healthy	#nra
#innovation	#metoo	#ddhh	#prolife
#blockchain	#notmypresident	#physical	#conservative
#digitalmarketing	#voteblue	#essays	#trump
#iot	#neveragain	#wildfire	#qanon
**Cluster e**	**Cluster f**	**Cluster g**	**Cluster h**
#bts	#blacklivesmatter	#writer	#ynwa
#army	#blm	#author	#lfc
#exo	#defenddaca	#actor	#jft96
#got7	#abolishice	#singer	#dontbuythesun
# 방탄소년단	#allblacklivesmatter	#producer	#lfcfamily
#jungkook	#translivesmatter	#poet	#6times
#nct	#nodapl	#songwriter	#sixtimes
#taehyung	#mmiw	#director	#liverpool
#jimin	#blueforsudan	#filmmaker	#liverpoolfc
#wannaone	#blacklivesmatter✊🏾	#editor	#allezallezallez
**Cluster i**	**Cluster j**	**Cluster k**	
#mufc	#music	#gamer	
#glazersout	#choir	#twitch	
#teamjesus	#acoustic	#ps4	
#luhg	#doggos	#twitchaffiliate	
#utfr	#rain	#xbox	
#woodwardout	#giggles	#streamer	
#mufc⚽	#series	#overwatch	
#kwankwasiyya	#newsong	#fortnite	
#oleout	#flute	#twitchkittens	
#martialfc	#slander	#ffxiv	

Table 6: Top-10 prototypical labels for bottom-level Louvain clusters with respect to the top (3rd) level, found above the 0.05 bit threshold defined in Figure 5, ordered by decreasing coherence. Each of these is presented with only its top-4 labels in Figure 2a, main text. Compare to the SBM-based labels in Tables 7 and Table 8.

Cluster 0 (d, 0.58, 0.49)	Cluster 1 (a, 0.34, 0.32)	Cluster 2 (f, 0.60, 0.57)	Cluster 3 (b, 0.42, 0.41)	Cluster 4 (c, 0.14, 0.10)
#maga	#devops	#blacklivesmatter	#jedifallenorder	#sbs
#kag	#azure	#blm	#dogoodrecklessly	#queenof
#trump2020	#aws	#translivesmatter	#safarilive	#hamont
#2a	#impinv	#transrightsarehumanrights	#fightwhitesupremacy	#osint
#prolife	#office365	#blacklivesmatter✊🏾	#ibelieveyou	#amajoche
#wwg1wga	#kubernetes	#tlm	#disarmhate	#mainbhichowkidar
#nra	#cloudnative	#muslimlivesmatter	#veteransresist	#savetheoa
#conservative	#trx	#ccsu	#equalrightsforall	#modifor2019
#tcot	#iiot	#dst1913	#nonazis	#newindia
#qanon	#sharepoint	#prayforsudan	#resist✊🏾	#babe
**Cluster 5 (h, 0.75, 0.73)**	**Cluster 6**	**Cluster 7**	**Cluster 8 (j, 0.40, 0.22)**	
#ynwa	#gotiges	#tamuc	#nasasocial	
#lfc	#sportsmarketing	#tmc🏁	#enigma	
#jft96	#internetmarketing	#mvrp	#happinessbegins	
#dontbuythesun	#customer	#freetheguys	#okstate21	
#lfcfamily	#econ	#rwtw	#sewing	
#sixtimes	#gogreek	#parody	#aotearoa	
#6times	#بريدة	#nsu🔰	#🇨🇦	
#redordead	#hackathon	#nsu20	#teamvixen	
#dontbuythes*n	#politas	#nsula	#aboriginal	
#ynwa🔴	#consumers	#futureeducator	#modi	

Table 7: Top-10 prototypical labels for level 3 SBM clusters with respect to SBM level 6, found above the 0.05 bit threshold defined in Figure 5, ordered by decreasing coherence. Compare to the Louvain-based labels, where markers such as (f, 0.60, 0.57) indicate the matched Louvain cluster in Table 6, its associated cluster overlap score (set intersection normalized by Louvain cluster cardinality), and the matching Jaccard index (set intersection normalized by the union of both sets). See §3 for why SBM levels 3 and 6 were selected to calculate coherence. Clusters between models are matched via overall cluster overlap maximization and implemented in the scipy library' s linear_sum_assignment function.

Cluster 0 (a, 0.58, 0.39)	Cluster 1 (f, 0.68, 0.39)	Cluster 2	Cluster 3 (c, 0.09, 0.05)	Cluster 4 (i, 0.27, 0.07)
#tech	#blacklivesmatter	#jucoproduct	#yeg	#tamuc
#music	#blm	#ncat	#edtech	#tmc🏁
#travel	#resist	#11	#mieexpert	#daddygang
#ai	#blackgirlmagic	#22	#siuc	#mvrp
#innovation	#notmypresident	#23	#nhpolitics	#freetheguys
#digital	#translivesmatter	#21	#appleteacher	#nevernation
#digitalmarketing	#blacklivesmatter✊🏾	#uwg	#newmusic	#rwtw
#technology	#transrightsarehumanrights	#txsu	#ownambassador	#parody
#fintech	#believewomen	#pvamu	#hmg	#beeznation
#iot	#protecttranslives	#24	#econdev	#nsu🔰

Cluster 5 (d, 0.58, 0.49)	Cluster 6 (h, 0.75, 0.47)	Cluster 7 (g, 0.40, 0.25)	Cluster 8	Cluster 9 (e, 0.53, 0.19)
#maga	#ynwa	#writer	#heatnation	#exo
#kag	#lfc	#author	#keeppounding	#got7
#trump2020	#jft96	#vegan	#vfl	#nct
#2a	#dontbuythesun	#filmmaker	#wethenorth	#day6
#prolife	#ggmu	#director	#fearthedeer	#wannaone
#wwg1wga	#lfcfamily	#plantbased	#gocougs	#findom
#nra	#sixtimes	#thriller	#thisismycrew	#superjunior
#conservative	#6times	#bestselling	#gobills	#bigbang
#tcot	#redordead	#vegan🌱	#savedthecrew	#ikon
#qanon	#dontbuythes*n	#editing	#rallytogether	#ripgranny

Cluster 10	Cluster 11	Cluster 12		
#foamed	#tmc	#bsu		
#ouat	#repealedthe8th	#umes		
#shadowhunters	#rip	#madridista		
#lucifer	#southernnotstate	#bsu21		
#riverdale	#aamu	#unt		
#agentsofshield	#nccu	#dsu		
#twu	#repealthe8th	#umesalumni		
#2018التواز_ام	#beyhive	#kfupm		
#ua	#wiu	#bbg		
#theoriginals	#odu	#أبي		

Table 8: Top-10 prototypical labels for level 4 SBM clusters with respect to SBM level 6, found above the 0.05 bit threshold defined in Figure 5, as in Table 7. Compare to Table 6 containing Louvain-based labels. Markers such as (f, 0.68, 0.39) indicate the matched Louvain cluster in Table 6, its associated cluster overlap score (set intersection normalized by Louvain cluster cardinality), and the matching Jaccard index (set intersection normalized by the union of both sets). See §3 for why SBM levels 4 and 6 were selected to calculate coherence. Clusters between models are matched via overall cluster overlap maximization and implemented in the scipy library's linear_sum_assignment function.

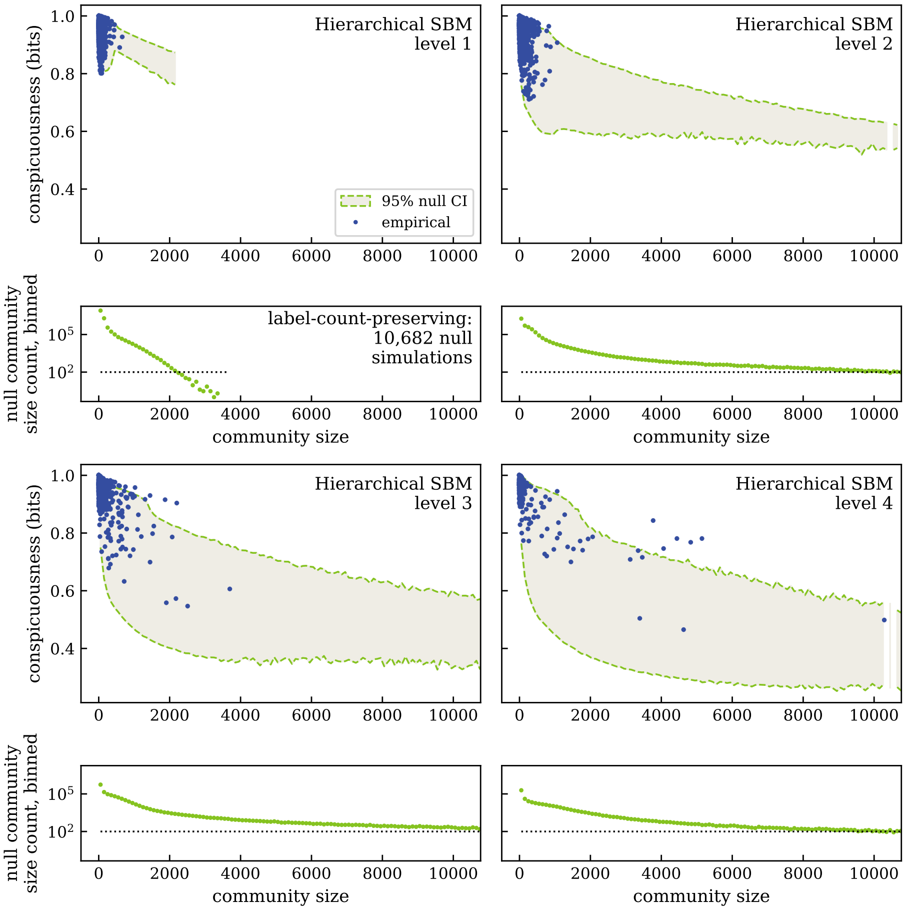

Figure 13: Conspicuousness results per lower SBM level, for comparison against the Louvain case in Figure 7 in this document and Figure 3 in the main text. The the 95% null CI bands for all four levels were generated from over 10,000 simulations using the same method as the Louvain case, replacing the Louvain algorithm with hierarchical SBM.

3.2 Conspicuousness at different SBM levels

We observe similar conspicuousness results for SBM as in the Louvain case (Figure 13). At the first SBM level, cluster size is curtailed overall in both empirical and null results (as discussed earlier in this document), and empirical conspicuousness is largely contained in the null region. As level increases beyond that base, we see results much more akin to our Louvain results (Figure 7 in this document and Figure 3 in the main text). Particularly by levels 3 & 4, we see conspicuousness diverging from null at larger cluster size–also observed in the Louvain-based analysis.

4 Hashtag glossary for main text

#coys “come on you spurs”: the Tottenham Hotspur Football Club

#ttp “trust the process” (in the sports context): refers to the long-term strategic coaching strategy of the Philadelphia 76ers

#mffl “Mave(erick)s fan for life”: Dallas basketball

#shsu, #shsu20 “Sam Houston State University”: university in Huntsville, Texas, home of the Bearkats

#maga, #kag : “make America great again”, “keep America great”: slogans for supporters of Donald Trump

#2a : Second amendment to the Constitution of the United States, the right to keep and bear arms

#fbr : “follow back resistance”: tag specifically meant to grow the anti-Trump #resistance movement through mutual followings on Twitter

#ai : artificial intelligence

#defenddaca : movement to retain Deferred Action for Childhood Arrivals

#abolishice : movement to abolish I.C.E., the US’ Immigration and Customs Enforcement

#blm : Black Lives Matter

#disneysmmc : Disney’s Mickey Mouse Club, a TV show

#ps4 : Playstation 4

#bts, #got7, #exo : K-pop bands

#army : here referring to the BTS fanbase

#mufc : Manchester United Football Club

#glazersout : Manchester United fan movement supporting removal of the Glazer family from club ownership

#teamjesus : in our figures, referring to Gabriel Fernando de Jesus, a football (soccer) player

#luhg : “Love United Hate Glazer”, related to #glazersout

#ynwa : “you never walk alone”, slogan used in football (soccer)

#lfc : London Football Club

#jft96 : “Justice for the 96”, in rememberance of 96 people crushed at a football match in 1996, the Hillsborough Disaster

#dontbuythesun : referring to the British tabloid The Sun

#orangenation : here referring to sports in Syracuse, New York

#jucoproduct : having to do with the junior college (JUCO), or community college level

#arsenal : a football club in the U.K.

#cfc : Chelsea Football Club

#coyg : “Come on you gunners”: fight song/phrase supporting Arsenal

#halamadrid : cheer for Real Madrid

References

[1] Jeff Alstott, Ed Bullmore, and Dietmar Plenz. powerlaw: A python package for analysis of heavy-tailed distributions. *PLOS ONE*, 9(1):1–11, 01 2014.

[2] Aaron Clauset, Cosma Rohilla Shalizi, and M. E. J. Newman. Power-law distributions in empirical data. *SIAM Review*, 51(4):661–703, 2009.

[3] Andreas Klaus, Shan Yu, and Dietmar Plenz. Statistical analyses support power law distributions found in neuronal avalanches. *PLOS ONE*, 6(5):1–12, 05 2011.

[4] Tiago P Peixoto. The graph-tool python library, 2014.

[5] Tiago P Peixoto. Hierarchical Block Structures and High-Resolution Model Selection in Large Networks. *Physical Review X*, 4(1):011047, March 2014.

[6] Tiago P Peixoto. Nonparametric weighted stochastic block models. *Physical Review E*, 97:012306, January 2018.

[7] Tiago P Peixoto. Bayesian stochastic blockmodeling. *Advances in network clustering and blockmodeling*, pages 289–332, 2019.

[8] Tiago P Peixoto. Revealing Consensus and Dissensus between Network Partitions. *Physical Review X*, 11:021003, April 2021.

[9] M Ángeles Serrano, Marián Boguñá, and Alessandro Vespignani. Extracting the multiscale backbone of complex weighted networks. *Proceedings of the National Academy of Sciences*, 106(16):6483–6488, April 2009.

[10] Lizhi Zhang and Tiago P Peixoto. Statistical inference of assortative community structures. *Phys. Rev. Research*, 2:043271, November 2020.
